# High Whey Protein Intake Delayed the Loss of Lean Body Mass in Healthy Old Rats, whereas Protein Type and Polyphenol/Antioxidant Supplementation Had No Effects

**DOI:** 10.1371/journal.pone.0109098

**Published:** 2014-09-30

**Authors:** Laurent Mosoni, Eva Gatineau, Philippe Gatellier, Carole Migné, Isabelle Savary-Auzeloux, Didier Rémond, Emilie Rocher, Dominique Dardevet

**Affiliations:** 1 INRA (Institut National de la Recherche Agronomique), UMR (Unité Mixte de Recherche) 1019 Nutrition Humaine, Saint Genès Champanelle, France; 2 Université Clermont 1, UFR (Unité de Formation et de Recherche) Médecine, UMR (Unité Mixte de Recherche) 1019 Nutrition Humaine, Clermont-Ferrand, France; 3 INRA (Institut National de la Recherche Agronomique), UR (Unité de Recherche) 370 QuaPA (Qualité des Produits Animaux), Saint-Genès Champanelle, France; 4 Danone Research, Palaiseau, France; Pennington Biomed Research Center, United States of America

## Abstract

Our aim was to compare and combine 3 nutritional strategies to slow down the age-related loss of muscle mass in healthy old rats: 1) increase protein intake, which is likely to stimulate muscle protein anabolism; 2) use leucine rich, rapidly digested whey proteins as protein source (whey proteins are recognized as the most effective proteins to stimulate muscle protein anabolism). 3) Supplement animals with a mixture of chamomile extract, vitamin E, vitamin D (reducing inflammation and oxidative stress is also effective to improve muscle anabolism). Such comparisons and combinations were never tested before. Nutritional groups were: casein 12% protein, whey 12% protein, whey 18% protein and each of these groups were supplemented or not with polyphenols/antioxidants. During 6 months, we followed changes of weight, food intake, inflammation (plasma fibrinogen and alpha-2-macroglobulin) and body composition (DXA). After 6 months, we measured muscle mass, in vivo and ex-vivo fed and post-absorptive muscle protein synthesis, ex-vivo muscle proteolysis, and oxidative stress parameters (liver and muscle glutathione, SOD and total antioxidant activities, muscle carbonyls and TBARS). We showed that although micronutrient supplementation reduced inflammation and oxidative stress, the only factor that significantly reduced the loss of lean body mass was the increase in whey protein intake, with no detectable effect on muscle protein synthesis, and a tendency to reduce muscle proteolysis. We conclude that in healthy rats, increasing protein intake is an effective way to delay sarcopenia.

## Introduction

Aging is characterized by a gradual loss of muscle proteins, also called sarcopenia, which has been well characterized both in humans [1] and in rodents [2]. This atrophy is associated with a progressive loss of muscle strength that directly affects mobility and health of elderly people. The origin of this phenomenon is multifactorial and can be the consequence of decreased physical activity, undernutrition, low grade inflammation, oxidative stress and endocrine changes.

Over the past years, consistent findings showed that one mechanism which could partly explain sarcopenia was a decreased ability of old muscle to respond appropriately to anabolic stimuli, such as food, amino acid, or leucine intake [3]–[8]: protein synthesis is less stimulated and protein degradation is less inhibited by amino acid intake in old muscle than in adult muscle, and more amino acids or more leucine are necessary to obtain a normal response in old muscle. Thus, a simple strategy to slow the age-related loss of muscle proteins would be to increase protein intake. An alternative strategy was proposed: use whey proteins as protein source. Indeed, whey proteins, are rapidly digested, leucine rich, and were shown, in the short term, to overcome anabolic resistance in old rats [9] as well as in humans [10]. They induce a rapid and robust rise in plasma amino acid levels after feeding, which can overcome muscle anabolic resistance.

The mechanism involved in this anabolic resistance is still unknown. Some data show that it is present in case of low-grade inflammation and absent when there is no inflammation [11], [12]. Oxidative stress could also be involved [13]–[17]. We showed previously that a 7 week supplementation with a polyphenol/antioxidant mixture containing rutin, vitamin E, vitamin A, zinc and selenium improved, in vitro, the anabolic response of old muscle to leucine [18]. Thus, beside increasing protein intake or using whey as protein source, a third strategy to limit the loss of muscle proteins during aging would be to limit inflammation and/or oxidative stress, for instance through supplementation with polyphenols and antioxidants.

To our knowledge, comparison or combination of these 3 strategies (increase in protein intake, use of whey as protein source, or polyphenol/antioxidant supplementation) was never tested before, in particular in the long term. An increase in protein intake seems a straightforward and efficient strategy. However, it could be difficult to increase protein intake in elderly subjects because their appetite is limited and proteins are highly satiating. In addition, in case of pre-existing renal alteration, protein intake must be limited. Finally, it is now more and more recognized that to be able to feed an increasing world population, we have to spare proteins [19]. Using whey proteins could be a way to keep a normal protein intake while having the beneficial effects of a higher protein intake. Supplementation with polyphenols/antioxidants could also spare proteins by restoring a normal anabolic response of old muscles.

Thus, in the present study performed in old rats, we compared casein fed rats (12% protein) supplemented or not with an anti-inflammatory/antioxidant mixture, whey fed rats (12% protein) supplemented or not with an anti-inflammatory/antioxidant mixture, and high protein whey fed rats (18% protein) supplemented or not with an anti-inflammatory/antioxidant mixture. This anti-inflammatory/antioxidant mixture was composed of chamomile extract, vitamin E, and vitamin D. Indeed, while vitamin E is a recognized antioxidant, chamomile extract is a source of apigenin and rutin (metabolized as quercetine). These flavonols have antioxidant and anti-inflammatory properties [20]–[22]. Vitamin D was also described recently as having anti-inflammatory effects [23] and seems to improve muscle function [24]. This nutritional experiment lasted 6 months, and we measured changes of rat body composition, oxidative and inflammatory status, muscle and organ weight, fed and post-absorptive muscle protein synthesis, both in vivo and in vitro, and in vitro muscle proteolysis. We aimed to know which strategy was most efficient to slow down the loss of lean body mass during aging. We showed that only an increase in protein intake could slow down the loss of lean body mass.

## Methods and Materials

### Ethics statement

This protocol was conducted prior to 2013 in accordance with French legislation at this time. This experiment could be legally performed because: 1) INRA is licensed by the French Ministry of Agriculture to perform experiments in rats and our animal facilities are licensed to house and maintain rats; 2) the primary researcher of this work, as well as all the persons that interacted with the animals hold a personal license from the French Ministry of Agriculture. All efforts were made to minimize suffering (which is very low in this type of nutritional protocol) and final euthanasia was performed under overdose of sodium pentobarbital.

### Animals and diet

We used 172 male Wistar rats (Centre d'élevage Janvier, Le Genest-St-Isle, France), which were fed experimental diets for 6 months from the age of 16 months until the age of 22 months. First, at the age of 13 months, rats were housed under standard breeding conditions for 2 month. Then, they were placed in collective cages (three rats per cage) with a 12-h dark period starting at 08:30 and accustomed to a daily feeding period from 08:30 to 16:00 during one month: pellets were distributed at 8:30 in the morning and removed at 16:00 every night. Food consumption was monitored. The same daily feeding period was used during the whole experiment. After one month, a blood sample was withdrawn from the tail vein of each rat to assess plasma fibrinogen, α2-macroglobulin and albumin levels (basal measurement). Body composition was also measured by DXA (Dual Energy X-ray Absorptiometry, Hologic QDR 4500A; Hologic Inc., Waltham, MA): animals remained fasted and were anesthetized (75 mg/kg of ketamine and 6.25 mg/kg acepromazine) just before measuring. Then, rats were allocated into 6 groups, with similar weight in each group (mean and SE were homogenous in each group: from 662 to 675 g for the mean, and 14 to 18 g for the SE) and similar body composition in each group (lean body mass: from 468±9 g to 473±12 g; fat mass: from 178±10 g to 186±11 g) and experimental diets were started. At this point, the age of rats was 16 months. The diets contained either 14% raw casein (Cas – 12% protein), 14% raw whey (Whey - 12% protein), or 21.5% raw whey (Whey HP – 18% protein) and were supplemented (Aox+) or not (Aox−) with anti-inflammatory/antioxidant (see detailed diet composition in Table 1). Polyphenol supplementation consisted of 20 g/kg diet of chamomile extract (substituted with 10 g/kg diet of cellulose and 10 g/kg diet of wheat starch). Using apigenine as a marker, we showed in preliminary experiments that this dose induced a significant increase in plasma apigenine in rat plasma (peak to 8 µM) during 24 h and was not high enough to reduce food intake or protein digestibility (unpublished data). We also increased vitamin E diet content from 71 to 300 UI/kg diet. This dose seemed effective and well tolerated [18]. Vitamin D diet content was increased from 1000 to 5000 UI/kg diet which can be considered as a low dose given orally (4 to 5 µg/kg) [25]. Diets were prepared by a specialized unit of INRA (UPAE, INRA, Jouy en Josas, France) and were given as pellets to the animals during 6 months.

**Table 1:** Diet composition.

	Casein		Whey		Whey HP	
	Aox−	Aox+	Aox−	Aox+	Aox−	Aox+
	*Components (g/kg diet)*					
Casein	140	140	0	0	0	0
Whey	0	0	140	140	215	215
L-cysteine	1.8	1.8	0	0	0	0
Tyrosine	0	0	2.3	2.3	0	0
Arginine	0	0	1.7	1.7	0	0
Rapeseed oil	20	20	20	20	20	20
Sunflower oil	2	2	2	2	2	2
Peanut oil	18	18	18	18	18	18
Mineral mixture*	35	35	35	35	35	35
Vitamin mixture*	10	10	10	10	10	10
Choline bitartrate	2.5	2.5	2.5	2.5	2.5	2.5
Chamomile extract	0	17	0	17	0	17
Lactose	100	100	86	86	79	79
Sucrose	127	127	127	127	127	127
Cellulose	50	40	50	40	50	40
Wheat starch	493.7	486.7	505.5	498.5	505.5	434.5
Protein	116	116	114	114	175	175
Leucine	10.1	10.1	13.8	13.8	21.1	21.1
Fat	42.1	42.1	41.4	41.4	42.2	42.2
Fibers	50	57	50	57	50	57
Energy –kcal/kg	3783	3755	3811	3783	3792	3764
Vitamin E - UI/kg	71	300	71	300	71	300
Vitamin D - UI/kg	1000	5000	1000	5000	1000	5000

*Mineral and vitamin mixtures were made according to AIN 93 except in Aox+ rats: in these groups, vitamin E and vitamin D content were increased to provide 300 and 5000 UI/kg diet respectively.

Casein diet was supplemented with cysteine according to AIN 93. We considered that the AIN 93 diet corresponded to the basal requirements of rats, thus whey diet was supplemented with tyrosine and arginine to provide as much of these two amino acids as the AIN 93 diet.

Food intake was monitored weekly. Animals were weighed once every two weeks. After 3 and 6 months of supplementation blood sampling was performed in a lateral tail vein to assess plasma fibrinogen and *α*2-macroglobulin albumin levels. Body composition was also measured a second time at the end of the 6 month supplementation period. Finally, in vivo muscle protein synthesis rates and ex-vivo muscle protein synthesis and degradation rates were measured either in the post-absorptive state (PA) for half of the rats (food was not given in the morning as usual and measurements were made within 3 h after the usual feeding time) or in the post-prandial state (PP - food was given as usual in the morning and measurements were made 2 h after feeding) for the other half. Thus, we had finally 12 groups of rats: Cas Aox− PA (n = 11), Cas Aox− PP (n = 11), Cas Aox+ PA (n = 11), Cas Aox+ PP (n = 11), Whey Aox− PA (n = 12), Whey Aox− PP (n = 11), Whey Aox+ PA (n = 10), Whey Aox+ PP (n = 10), Whey HP Aox− PA (n = 11), Whey HP Aox− PP (n = 13), Whey HP Aox+ PA (n = 11) and Whey HP Aox+ PP (n = 10).

### Measurement of in vivo muscle protein synthesis rates

#### Tracer injection and tissue collection

Protein synthesis was assessed in vivo in the gastrocnemius muscle with the flooding dose method as described previously [26]. Briefly, 20 min before killing, each rat was injected intravenously with L-valine (150 µmol/100 g body weight) containing 50% of L-[1-13C] valine (Euriso-Top, Saint Aubin, France). Animals were then anesthetized with pentobarbital sodium (6 mg/100 g body weight). Epitrochlearis muscles were quickly excised and incubated ex-vivo for proteolysis measurement. Blood was collected from abdominal aorta and centrifuged at 3000 *g* for 10 min (+4°C). Posterior leg skeletal muscles (gastrocnemius, tibilalis anterior, soleus and extensor digitorum longus), fat tissues, and organs (liver, heart, kidneys and spleen) were excised, weighed, frozen in liquid nitrogen and stored at −80°C until analysis.

#### Measurements of valine enrichments

Free and protein bound valine enrichments were determined as described previously [27]. Briefly, gastrocnemius muscle was powdered in liquid nitrogen in a ball mill (Dangoumeau, Prolabo, Paris, France). A 0.2 g aliquot of frozen muscle powder was homogenized in 8 volumes of ice-cold 0.61 mol/L trichloroacetic acid (TCA) (Potter, Bosch, France). Homogenates were centrifuged (5000× g, 15 min, 4°C) and supernatants, containing free amino acids, were desalted by cation-exchange chromatography (AG 50×8, 100–200 mesh, H+ form Bio-Rad, Richmond, CA) in mini-disposable columns. Amino acids were eluted with 4 mol/L NH_4_OH. After evaporation of NH_4_OH under vacuum, free amino acids were suspended in 0.01 mol/L HCl for enrichment measurements. TCA-insoluble materials were washed in 4 volumes of ice cold 0.61 mol/L TCA and 3 times in 4 volumes of 0.2 mol/L perchloric acid (HClO4). Resultant pellets were solubilized in 0.3 M NaOH acid (Sigma Aldrich, Chesnes, France), incubated at 37°C for 1 h, and protein concentration was determined in an aliquot using bicinchoninic acid [28]. Proteins were precipitated again with 1.99 mol/L HClO4 overnight at 4°C, the samples were centrifuged (10,000× g, 5 min, 4°C), and the protein pellet was hydrolyzed in 6 mol/L HCl at 110°C for 48 h. HCl was removed by evaporation and amino acids purified by cation-exchange chromatography as described above. Measurement of free valine enrichment was done as its t-butyldimethylsilyl derivative under electron impact ionization by gas chromatography/mass spectrometry (GC-MS), with an HP-5890 gas chromatograph coupled to an HP-5972 organic quadrupole mass spectrometer (Hewlett-Packard, Paris, France). The ions m/z 288 and 289 were monitored by selective ion recording to determine the [^13^C]valine enrichment. Enrichment of [^13^C]valine into protein was measured as its N-acetyl-propyl derivative by gas chromatography-combustion-isotope ratio mass spectrometry (GC-C-IRMS, Micromass Isochrom II, Fisons Instruments, Middlewitch, UK) which measured the ratio ^13^CO_2_/^12^CO_2_.

#### Calculations

In vivo Fractional Synthesis Rates (FSR, %.d) of tissue proteins were calculated as described previously [3]: FSR = 100×(EP-EN)/(EA×t) where t is the incorporation time, expressed in days, EP and EA are the ^13^C enrichments of protein-bound valine and of free valine respectively, at the end of the incorporation time. Incorporation time was measured for each rat between the time of injection and the time of exsanguination and averaged 23.5±2.3 min (means ± SE). EN is an estimation of the natural 13C enrichment of protein-bound valine. It was determined in 3 rats which were not injected with the flooding dose. EP, EN and EA were expressed in AP (atom %).

### Measurement of in vitro protein synthesis and proteolysis

Muscle protein synthesis was measured ex-vivo in epitrochlearis muscles as described previously [18] 1) in fed and post-absorptive rats (effect on in vivo feeding); 2) in post-absorptive rats in presence (left muscle) or absence (right muscle) of 200 mM leucine in incubation medium: this constitute a very good test of the sensitivity of muscle protein synthesis to feeding. Epitrochlearis muscles were preincubated for 30 min at 37°C in Krebs-Henseleit buffer (containing (mM): 120 NaCl, 4.8 KCl, 25 NaHCO3, 2.5 CaCl2, 1.2 KH2PO4 and 1.2 MgSO4; pH 7.4), supplemented with 5 mM HEPES, 5 mM glucose and 0.1% bovine serum albumin (saturated with 95% O_2_-5% CO_2_). Muscles were then transferred into the same medium containing 0.5 mM L-[^14^C] phenylalanine (0.15 µCi/ml) and incubated under the same conditions for 1 h.

At the end of incubation, muscles were blotted, weighed, homogenized in 10% trichloroacetic acid (TCA) and centrifuged at 10 000 g for 10 min at 4°C. Pellets were washed 3 times with 10% TCA and solubilized in 1N NaOH at 37°C for determination of protein content and protein-bound radioactivity. Protein-bound radioactivity was measured using liquid scintillation counting. Protein synthesis was calculated by dividing protein-bound radioactivity by specific activity of phenylalanine in incubation medium. It was expressed as nanomole phenylalanine incorporated per milligram protein per hour [18].

Proteolysis was determined as described previously [18] by tyrosine accumulation into incubation medium in fed and post-absorptive rats. Because tyrosine is neither synthesized nor degraded in muscle, the release of tyrosine into the incubation medium directly reflects net protein breakdown. Proteolysis was thus estimated at the same time as protein synthesis by summing net tyrosine release and tyrosine incorporation into proteins (after conversion of the rate of phenylalanine incorporation into proteins into tyrosine equivalents as previously described [29]. Tyrosine concentration was determined using fluorometry according to [30]. Protein degradation was expressed in nanomole tyrosine produced per milligram protein per hour.

### Inflammatory status and oxidative status

Plasma fibrinogen was measured by turbidimetry on a Cobas Mira analyzer. Plasma *α*2-macroglobulin was measured by single radial immunodiffusion [31]. Total glutathione content was measured in liver and muscle after extraction of an aliquot in 0.2 M PCA and 5 mM EDTA solution as described previously [32]. Tissues superoxide dismutase activity was also measured in liver and muscle after extraction in phosphate buffer using RANSOD kit (Randox laboratories, Montpellier, France). The same extract was used for antioxidant capacity assay with TAS kit (Randox laboratories, Montpellier, France). Muscle carbonyl content was measured spectrophotometrically after reaction between soluble proteins and dinitrophenyl hydrazine as described previously [33].

### Statistical analysis

Data were analyzed by variance analysis using SAS software (CARY, NC, USA). We used a three way variance analysis: 1) “protein nutrition” (Cas, Whey); 2) protein intake level (12%, 18%); 3) antioxidant intake (Aox+, Aox−). We also analyzed our results using a two-way variance analysis: 1) “protein nutrition” (Cas 12%; Whey 12%; Whey 18%); antioxidant intake (Aox+, Aox−). Both statistical models gave the same results. We present here results of the three-way variance analyses. In addition, it was necessary for some measurements (fractional synthesis rates for instance) to add “nutritional state” (post absorptive and postprandial) as explicative variable. For body weight and food intake, we performed repeated time variance analysis. Data are expressed as means ± SE. The level of significance was set at *P*<0.05.

## Results

### Food intake, weight, body composition and tissue weights

**Figure 1:**
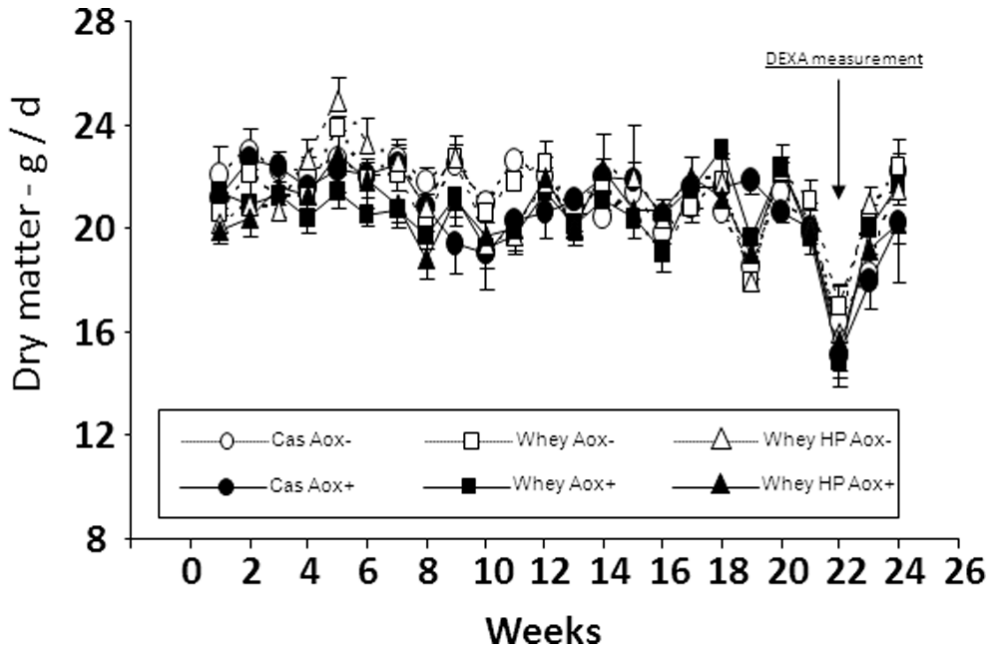
Changes of food intake in all groups during the experimental period. Six groups were compared: casein fed rats without anti-inflammatory/antioxidant supplementation (cas Aox−); casein fed rats with anti-inflammatory/antioxidant supplementation (cas Aox+); whey protein fed rats without anti-inflammatory/antioxidant supplementation (Whey Aox−); whey protein fed rats with anti-inflammatory/antioxidant supplementation (Whey Aox+); whey protein fed rats without anti-inflammatory/antioxidant supplementation with a high protein content (18% vs 11–12% in other groups) (Whey HP Aox−); whey protein fed rats with anti-inflammatory/antioxidant supplementation with a high protein content (18% vs 11–12% in other groups) (Whey HP Aox+). These different diets were given during 6 months. Food intake fluctuated with time without any discernible trend. Overall, food intake was not significantly different between groups.

**Figure 2:**
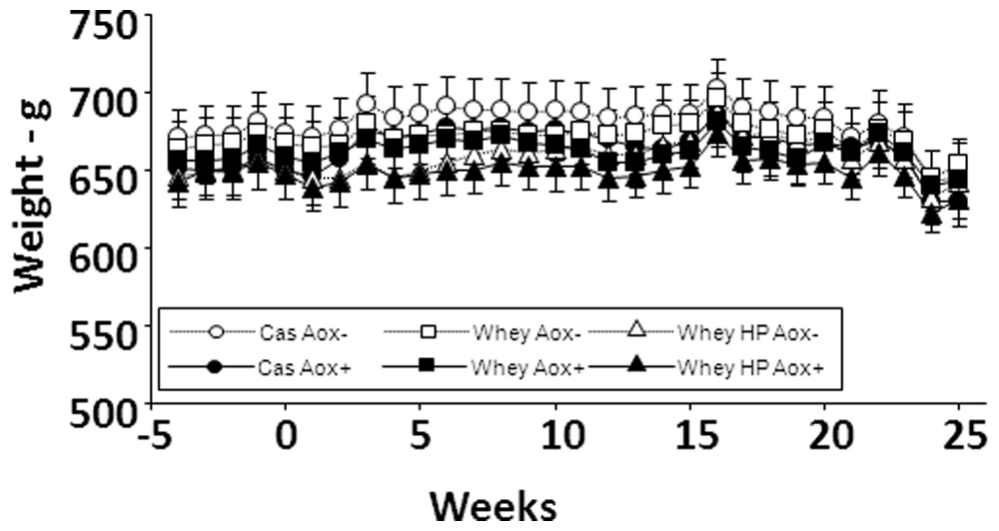
Changes of body weight in all groups during the experimental period. See figure 1 for group description. Body weight fluctuated with time without any discernible trend. Overall, body weight was not significantly different between groups.

Mean food intake averaged 20.9±0.3 g/day. Food intake fluctuated over time (P<0.01), but without any discernible trend. Overall food intake was not different between groups (Figure 1). Similarly, although body weight fluctuated over time, overall, there was no significant difference between groups (Figure 2, Table 2). However, lean body mass significantly decreased and fat mass, as well as percent fat mass, significantly increased during the nutritional experiment (Table 2). Variance analysis showed that protein source (Cas vs Whey) and antioxidant supplementation (Aox− vs Aox+) had no effects on these age-related modifications. However, protein level (12% vs 18%) had a significant effect. The loss of lean body mass was lower in 18% protein fed animals than in 12% protein fed animals (Table 2). This effect of protein level was not detected when comparing tissue weight in all groups at the end of the experimental period (Table 3). In particular, there were no significant differences between groups for muscle weights (Table 3). Significant differences in liver and kidney weights in anti-inflammatory/antioxidant supplemented animals were observed (Table 3).

**Table 2:** Evolution of body composition during the experimental period.

	Cas		Whey		Whey HP	
	Aox−	Aox+	Aox−	Aox+	Aox−	Aox+
**Body W**			g			
Before	660±19	642±20	649±16	642±14	638±13	641±18
After	653±20	637±15	645±17	634±16	641±12	642±14
**LBM**			% body weight			
Before	71.5±1.3	72.8±1.6	72.4±1.4	72.6±1.3	73.6±0.9	72.9±1.0
After	69.7±1.6	69.0±1.6	69.9±1.6	69.5±1.3	71.2±0.7	70.7±1.2
			↓* - g			
Before	469±11	462±11	468±8	464±9	468±9	466±13
After	450±10	437±10	446±6	438±10	455±7	451±9
% change	−3.7±1.5	−5.3±1.0	−4.4±0.7	−5.5±1.3	−2.6±0.9†	−2.5±1.2
**Fat mass**			↑* - % body weight			
Before	28.5±1.3	27.2±1.6	27.6±1.4	27.4±1.3	26.4±0.9	27.2±1.0
After	30.6±1.6	31.0±1.6	30.1±1.6	30.5±1.3	28.9±0.7	29.3±1.2
			↑* - g			
Before	191±13	179±15	183±14	178±11	170±8	175±9
After	203±15	200±12	200±16	196±12	186±7	190±11
% change	+7.8±5.9	+18.0±6.5	+11.3±5.7	+12.9±5.1	+12.7±4.5	+11.0±4.9

“Body W” is body weight, “LBM” is lean body mass, “% change” is the modification of total lean body mass or total fat mass during the experimental period. ↓*: Main effect “time” of variance analysis was significant for lean body mass expressed in g (P<0.01). ↑* Main effect “time” of variance analysis was significant for fat mass expressed in g and in % body weight (P<0.01).

† Main effect “protein level” of variance analysis was significant for % change of lean body mass (P = 0.03).

Regarding post hoc mean comparisons, there was never a significant difference between means with P<0.05.

**Table 3:** Comparison of tissue weights in nutritional groups at the end of the experimental period.

	Cas		Whey		Whey HP	
Weight	Aox−	Aox+	Aox−	Aox+	Aox−	Aox+
			*g or mg*			
Gastrocnemius	4.40±0.17	4.60±0.16	4.75±0.16	4.68±0.16	4.64±0.18	4.80±0.19
Tibialis ante.	1.33±0.06	1.35±0.05	1.43±0.05	1.40±0.06	1.38±0.05	1.42±0.06
EDL - mg	457±15	449±13	463±14	469±15	451±18	459±15
Soleus - mg	444±20	497±16	470±19	465±21	454±25	463±29
Sum	6.63±0.25	6.90±0.22	7.12±0.22	7.02±0.23	6.92±0.25	7.14±0.27
Liver*	15.6^a^ ^b^±0.7	17.5^c^±0.8	15.2^a^ ^b^±0.4	17.6^c^±0.4	14.7^b^±0.5	16.7^a^ ^c^±0.6
Kidneys*	3.7^a^ ^b^±0.2	4.0^a^±0.2	3.4^b^±0.1	3.8^a^ ^b^±0.1	3.6^b^ ^c^±0.1	3.8^a^ ^c^±0.1
Spleen	1.37±0.08	1.32±0.06	1.42±0.05	1.32±0.04	1.35±0.06	1.29±0.05
Heart	1.71±0.06	1.72±0.04	1.73±0.06	1.69±0.03	1.71±0.05	1.71±0.04
Perirenal AT	19.5±1.6	17.4±1.0	19.7±1.7	19.4±1.3	18.8±1.3	16.4±1.2
Perigenital AT	15.5±1.2	14.7±1.4	15.5±1.1	15.3±0.7	15.7±0.8	13.4±0.9

«Tibialis ante.» = tibialis anterior; «EDL» = extensor digitorum longus; «AT» = adipose tissue.

*: The only significant effects were: effect of antioxidant supplementation on liver weight (P<0.01) and on kidney weight (P<0.05).

a, b, c : mean values affected with the same letter were not significantly different.

### Mortality, inflammation, oxidative stress

There were no differences between groups in mortality during the 6 month experimental period (Number of deaths: Cas Aox−: 6; Cas Aox+: 7; Whey Aox−: 6; Whey Aox+: 8; Whey HP Aox−: 4; Whey HP Aox+: 8).

**Figure 3:**
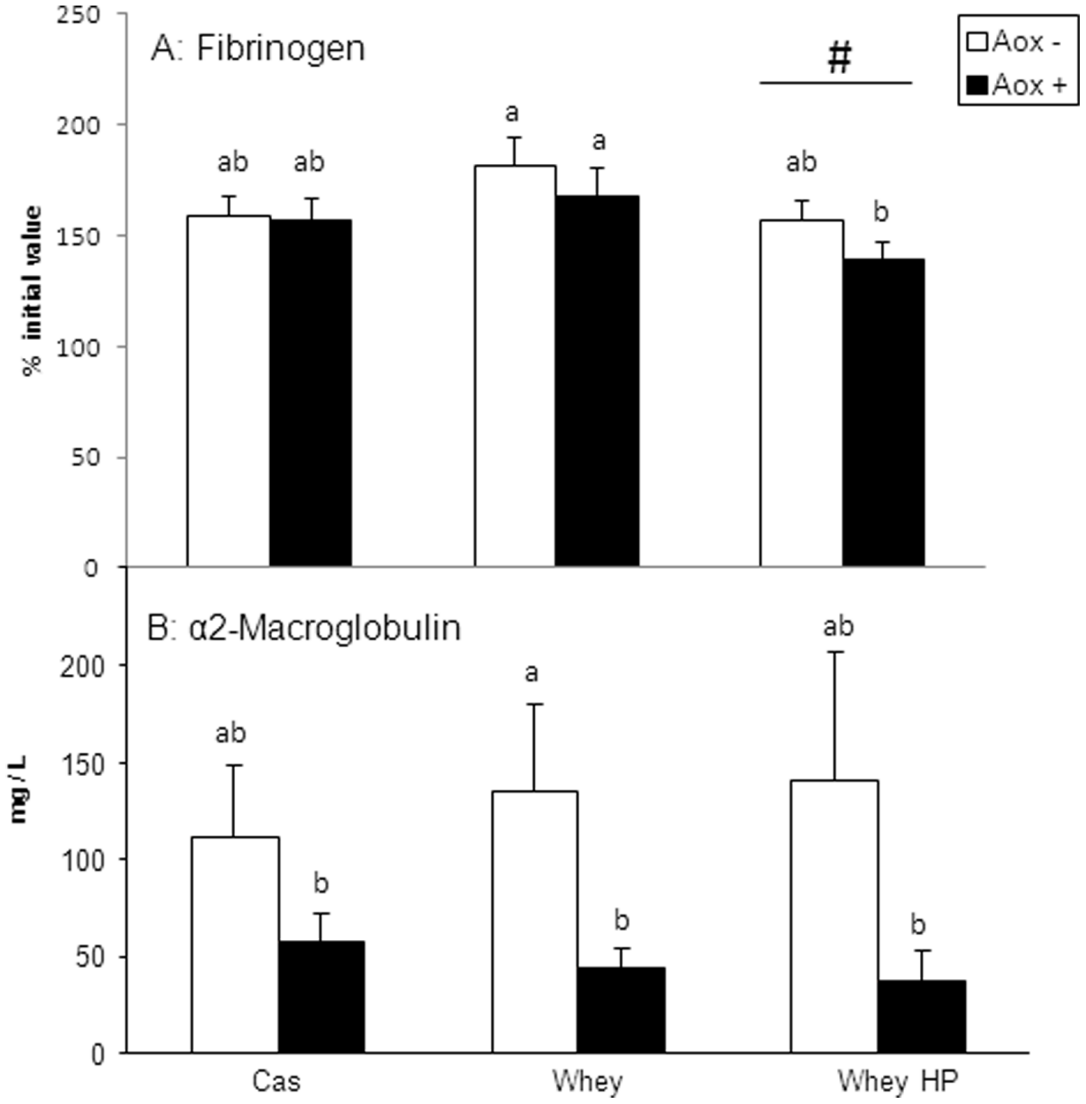
Fibrinogen and α2-macroglobulin plasma levels at the end of the experimental period. See figure 1 for group description. a,b,c…: means affected with the same letters were not significantly different. A: Fibrinogen values are expressed in % of the value measured at the beginning of the experiment. #: main effect “protein level” of variance analysis was significant (P = 0.04). B: α2-macroglobulin values are expressed in mg/L. Variance analysis detected a trend (P = 0.09) for main effect “anti-inflammatory/antioxidant supplementation”.

At the beginning of the experimental period plasma fibrinogen content averaged 2.36±0.04 g/l for all rats with no differences between groups. After 3 months, there were still no differences between groups, and the population mean value (2.60±0.05 g/l) increased slightly (10%) but significantly (P<0.01). After 6 months, mean value increased by 55%, averaging 3.67±0.08 g/l. This increase was significantly lower in the Whey HP group than in the other groups (P for level of protein X time interaction = 0.04) (Figure 3A). However, this increase was not affected by anti-inflammatory/antioxidant supplementation or by the nature of ingested protein (Figure 3A).

Regarding α2-macroglobulin, plasma values obtained at the beginning and in the middle of the experiment were too low to be detected (<10 µg/mL). It was only after 6 months that these values could be measured. Thus, at the end of the experimental period, variance analysis detected a trend (P = 0.09) for a decrease in plasma α2-macroglobulin level in rats supplemented with anti-inflammatory/antioxidants (Figure 3B). However, using a different statistical model with main effect “protein” (Cas, Whey and Whey HP), and main effect anti-inflammatory/antioxidant supplementation (Aox−, Aox+), polyphenol antioxidant supplementation was detected as significant (P = 0.006).

Among measured oxidative stress parameters, few parameters were significantly affected by nutritional treatments (Table 4). Anti-inflammatory/antioxidant supplementation significantly reduced muscle thiobarbituric acid reactive substances (TBARS) content, increased liver glutathione content, and increased plasma antioxidant activity (Table 4). Curiously, casein fed rats had also a higher liver SOD activity than Whey fed rats (Table 4). All other parameters were modified neither by the level of protein intake, the polyphenol/antioxidant content nor by the nature of ingested protein (Table 4).

**Table 4:** Oxidative stress parameters in gastrocnemius muscle, liver and plasma at the end of the nutritional period.

	Cas		Whey		Whey HP	
	Aox−	Aox+	Aox−	Aox+	Aox−	Aox+
**Muscle**						
Glutathione	0.63±0.04	0.64±0.04	0.69±0.03	0.69±0.03	0.69±0.03	0.72±0.03
SOD activity	6.0±0.3	5.5±0.2	5.7±0.3	5.5±0.4	5.8±0.2	5.8±0.2
TAC	0.16±0.03	0.13±0.01	0.15±0.02	0.12±0.02	0.17±0.03	0.13±0.02
Carbonyls	2.60±0.09	2.56±0.06	2.64±0.10	2.56±0.06	2.60±0.08	2.53±0.10
TBARS*	0.79^a^±0.08	0.65^b^±0.05	0.70^a^ ^b^±0.05	0.56^b^±0.03	0.71^a^±0.05	0.70^a^ ^b^±0.05
**Liver**						
Glutathione*	6.4^a^±0.2	9.0^b^±0.4	6.3^a^±0.2	9.1^b^±0.4	6.2^a^±0.3	8.8^b^±0.3
SOD activity†	17.9^a^±1.0	17.5^a^±0.7	16.0^a^ ^b^±0.9	14.5^b^±0.7	15.0^b^±0.6	14.7^b^±0.9
TAC	0.96±0.08	0.91±0.08	0.92±0.09	0.81±0.08	0.86±0.06	0.92±0.08
**Plasma**						
TAC*	0.88^a^±0.03	0.98^b^±0.03	0.89^a^±0.02	0.97^b^±0.03	0.92^b^ ^b^±0.03	0.98^b^±0.03

Glutathione content was expressed in µmoles/g tissue, SOD activity in U/mg protein, TAC ( = total antioxidant capacity) in moles of trolox equivalent/g protein, carbonyls in nmoles DNPH/mg protein, TBARS (thiobarbituric acid-reactive substances) in mg/kg tissue. Variance analysis detected a significant effect of *polyphenol/antioxidant supplementation (P<or = 0.01);

† : protein nature (casein vs Whey)(P<0.01).

a,b : mean values affected with the same letter were not significantly different.

### Protein metabolism

In vivo gastrocnemius muscle protein synthesis rates were stimulated by feeding (P = 0.045), but there was no effect of polyphenol/antioxidant supplementation, no difference between casein and Whey, and no difference between 12 and 18% protein (Table 5). The effect of feeding could only be detected by variance analysis: there was never a significant difference between groups in post-hoc statistical analysis (Table 5). Similar results were obtained regarding ex-vivo epitrochlearis muscle protein synthesis rates (Table 5): feeding was the only significant effect detected (P = 0.01). The lowest mean values were obtained in the Cas Aox− PA and Whey HP Aox+ PA groups (Table 5). We also tested the effect of leucine on ex-vivo epitrochlearis muscle protein synthesis rates. Leucine was able to stimulate significantly protein synthesis in all groups (P<0.001), but this stimulation was not modulated by polyphenol/antioxidant supplementation (Table 5). However, variance analysis detected a significant effect of protein type (P = 0.04): ex-vivo protein synthesis rates were higher with Whey than with caseins, but this was not detected when evaluating PA/PP effects. Finally, ex-vivo epitrochlearis muscle proteolysis rates were unaffected by feeding, polyphenol/antioxidant supplementation or protein type (Table 5). However, there was a trend (P = 0.09) towards reduced proteolysis in 18% protein fed rats compared to 12% protein fed rats (Table 5). This effect of “protein level” reached significance (P = 0.03) when only “protein level” and “nutritional state” were included as main effects in variance analysis.

**Table 5:** Muscle protein metabolism at the end of the nutritional period.

	Cas		Whey		Whey HP	
	Aox−	Aox+	Aox−	Aox+	Aox−	Aox+
**In vivo S**						
PA	6.67±0.43	6.23±0.29	6.18±0.28	6.30±0.44	6.35±0.26	5.95±0.21
PP	6.87±0.36	7.05±0.46	6.86±0.46	6.32±0.27	6.98±0.31	6.07±0.41
**Ex-vivo S**						
PA	0.31±0.01	0.36±0.02	0.35±0.02	0.36±0.03	0.36±0.02	0.33±0.01
PP	0.40^1^ ^2^±0.02	0.35±0.02	0.39^1^ ^2^±0.02	0.38^1^±0.02	0.38^1^±0.02	0.37±0.05
0 mM leu	0.28^4^±0.01	0.28^4^±0.01	0.32^3^±0.01	0.32^3^±0.02	0.31^4^±0.01	0.31^4^±0.01
200 mM leu	0.33^3^±0.02	0.33^3^±0.02	0.32^4^±0.01	0.34^3^±0.01	0.36^3^±0.02	0.35^3^±0.01
**Ex-vivo P**						
PA	1.33±0.08	1.26±0.06	1.30±0.07	1.29±0.04	1.22±0.04	1.23±0.05
PP	1.36±0.08	1.27±0.10	1.29±0.08	1.23±0.08	1.18±0.06	1.17±0.08

Values are expressed in means ± SE. PA = post-absorptive state. PP = post-prandial state. 0 or 200 mM leu: epitrochlearis muscles were incubated in medium containing either 0 or 200 mM leucine. **In vivo S** = in vivo gastrocnemius muscle fractional synthesis rates, expressed in %/day; the only significant effect detected by variance analysis was the effect of feeding (P = 0.045); however, there was no significant differences between means. **Ex-vivo S** = ex-vivo epitrochlearis muscle synthesis rates, expressed in µmoles phenylalanine/mg prot/h; experiment 1, PA/PP states: the only significant effect detected by variance analysis was the effect of feeding (P = 0.01);

1 = significantly different from Cas Aox− PA mean value;

2 = significantly different from Whey HP Aox+ PA; experiment 2, 0 or 200 mM leu: the only significant effect detected by variance analysis were the effect of leucine (P<0.001) and protein type (P = 0.04);

3 = significantly different from Cas Aox− 0 mM leu and Cas Aox+ 0 mM leu;

4 = significantly different from Whey HP Aox− 200 mM leu and Whey HP Aox+ 200 mM leu;

**Ex-vivo P** = ex-vivo epitrochlearis muscle proteolysis rates, expressed in µmoles tyrosine/mg prot/h; variance analysis showed that only protein level modulated proteolysis rates (Whey HP vs all other groups); it reached significance (P = 0.03) when only nutritional state and protein level were included in the model; there was no significant differences between means.

**Figure 4:**
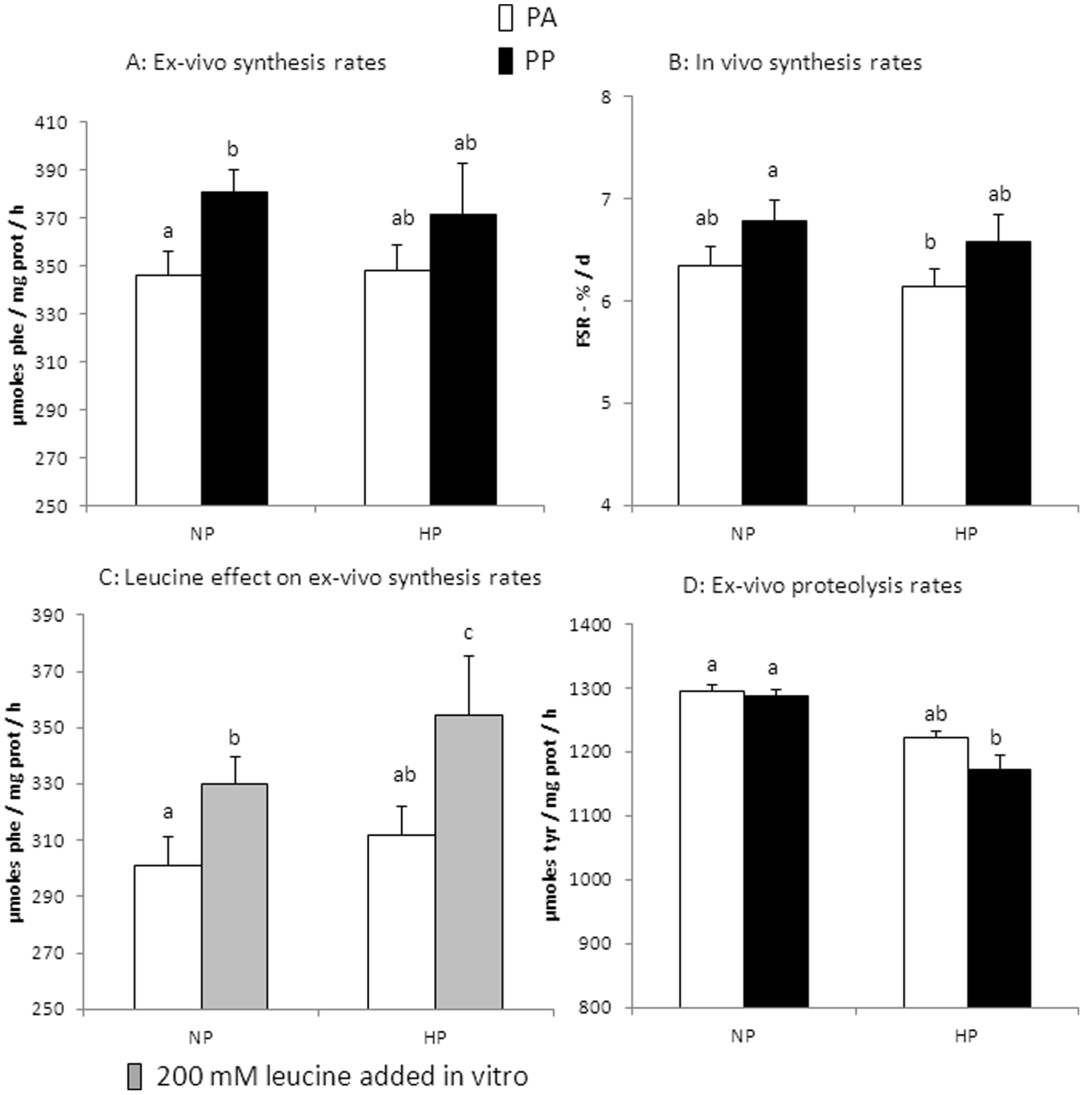
Focus on the effect of the level of protein intake on muscle protein metabolism. (see Table 5 for detailed data per group). We pooled data to focus on the effect of the level of protein intake to analyze how modifications of muscle protein metabolism could be in accordance with the fact that increasing whey protein intake slowed the age-related loss of lean body mass. NP: diet contained 12% casein or whey protein; HP: diet contained 18% whey protein; PA = post-absorptive state; PP: post prandial state. a,b,c…: means affected with the same letters were not significantly different. A: ex-vivo epitrochearis muscle protein synthesis rates (µmoles phenylalanine incorporated per mg protein and per hour). B: in vivo gastrocnemius muscle protein synthesis rates (fractional synthesis rates, %/day). C: leucine effect on ex-vivo epitrochlearis muscle protein synthesis rates (µmoles phenylalanine incorporated per mg protein and per hour). D: ex-vivo epitrochlearis muscle proteolysis rates (µmoles tyrosine released per mg protein and per hour). A, B, C: Variance analysis detected a significant effect of feeding (P<0.05). It was “protein level” for D (P = 0.03 if only “protein level” and “nutritional state” were included as main effects in variance analysis). See Table 5 for detailed group comparisons.

Since only an increase in protein level could slow down the age-related loss of lean body mass, we wondered in this effect could be explained by variations in protein metabolism. Complete results were given in Table 5, but to focus the presentation on the effect of high protein feeding and because there was no significant effect detected, Aox−, Aox+, Cas and Whey groups were pooled in Figure 4. Thus, we compared data obtained in 12% protein fed rats (NP) and 18% protein fed rats (HP) (Fig. 4). We see that protein synthesis is not higher in HP rats than in NP rats, and that feeding stimulation of muscle protein synthesis is also not different between HP rats and NP rats (Fig. 4). The only parameter that could explain the positive effect of high protein intake was lower proteolysis rates in HP rats than in NP rats (Fig. 4).

## Discussion

Our aim was to compare and combine nutritional strategies to limit the loss of muscle mass during aging. Three strategies were used: 1) increased whey protein intake; 2) use of leucine rich rapidly digested proteins i.e. whey proteins; 3) supplementation with anti-inflammatory/antioxidants. An increase in protein intake can be difficult (satiating effect, pre-existing renal alterations, shortage of proteins at the world level). The use of whey as protein source and/or polyphenol/antioxidant supplementation could be interesting alternatives. However, we showed that at normal protein intake, there was no difference between casein and whey, that polyphenol/antioxidant supplementation had no effects, and that finally, only an increase in whey protein intake delayed the loss of lean body mass.

Inflammation and oxidative stress are suspected to initiate anabolic resistance to feeding [12]. Previously, we showed that polyphenol/antioxidant supplementation improved muscle ex-vivo protein synthesis response to leucine [18] and improved oxidative stress and inflammation status [34]. Recently, it was shown that long-term supplementation with F1, a cystine-based glutathione precursor was able to delay loss of muscle mass in aged mice [35]. Another polyphenol, resveratrol, also in aged mice, did not delay loss of muscle mass but reduced muscle oxidative stress and preserved fast-twitch fiber contractile function [36], and improved muscle mass in hind limb suspended old rats [37]. Thus, it is clear that antioxidant/polyphenol supplementation can improve muscle function and muscle mass during aging (see also [14]). It is thus surprising that we did not detect more positive effects of our polyphenol/antioxidant supplementation in the present experiment. It may be related to the fact that the rats were particularly healthy: 1) absolute values for plasma fibrinogen in the beginning of our experiment (2.36±0.04 g/l) were significantly lower (P<0.01) than in our previous experiment (4.35±1.5 g/l – ref. [18]); 2) alpha2-macroglobulin was undetectable in the plasma of our rats at the beginning of the experiment and was only 79±12 mg/l at the end of the experiment whereas it averaged 186±42 mg/l previously [18]; 3) liver glutathione level (7.6±0.2 µmol/g) was also significantly higher (P<0.01) than in our previous experiment (4.6±0.2 µmol/g). This oxidative and inflammatory status was thus very good. It slightly deteriorated over the 6 months of experiment and anti-inflammatory/antioxidant supplementation had positive effects on oxidative stress (plasma antioxidant capacity, liver glutathione content, muscle TBARS) and inflammation (α2-macroglobulin). However, this supplementation did not affect changes of lean body mass nor muscle protein synthesis rates. Thus, we show here that in very healthy rats, polyphenol/antioxidant supplementation above nutritional recommendations, although improving oxidative stress and inflammation, has no effect on muscle protein metabolism.

Regarding our second strategy, replacing casein by whey proteins had also no effects on lean body mass, muscle protein synthesis and degradation rates. This result is in accordance with recent long term studies in rats [38] and in humans [39], and is also consistent with studies showing that long-term leucine supplementation had no effects on lean body mass or muscle mass in rats [40], [41] and in humans [42], [43]. However, studies are also in agreement to show that acute supplementation with leucine or whey proteins restore stimulation of muscle protein synthesis in old rats [9] or elderly humans [44]. There is thus a discrepancy between short term and long-term effects of leucine/whey proteins on muscle metabolism. It is possible that stimulation of protein synthesis is increased, but that this stimulation is short term so that protein synthesis returns quickly to post-absorptive levels. To solve this issue, it would be useful to follow protein synthesis over the hours following the meal. In any case, we showed here that long term replacement of casein by whey in healthy old rats had no effects not only on protein synthesis and degradation rates, but also on lean body mass and muscle mass.

Finally, the only strategy that had a significant effect on lean body mass was an increase in protein intake. We could detect a significant loss of lean body mass over 6 months in our healthy rats, and an increase in protein intake was able to slow down this age-related loss. We used whey proteins to increase protein intake, and it is not to be excluded that these proteins could have been more efficient than casein would have been, in particular due to their higher leucine content (12% casein = 10.1 g leucine/kg diet; 12% whey = 13.8 g leucine/kg diet; 18% whey = 21.1 g leucine/kg diet) However, we believe that the difference in the effects of these two proteins decreases with higher protein intake. May be, 20% or 23% casein would have been necessary to obtain the same effect (24% casein would have provided the same amount of leucine than 18% whey). In any case, the 18% whey diet could be seen as a model of “high protein diet”. Surprisingly, few studies tested the effect of increasing protein intake on the sparing of lean body mass during aging in the rat. In humans, many studies suggest a positive effect of protein or amino acid supplementation on lean body mass either in healthy [45], [46], frail [47], [48], or glucose intolerant [49] elderly subjects, even if few studies found no effects [50].

In our study, despite the preservation of lean body mass following 6 months of increased protein intake, we did not observe an effect on hind limb muscle mass. Since the effect on lean body mass is modest, it is possible that intra-group variability masked a potential effect on muscle mass. Indeed, effect on hind limb muscle mass could only be assessed at the end of the intervention thus preventing us from assessing the % of change over a 6 months period as was done for lean body mass. Beneficial effects of amino acid supplementation on muscle protein content or fiber area were observed previously [51], [52]. In addition, high protein diets improved muscle recovery after immobilization in old rats [53]. Thus, the positive effect of increased protein intake that we observed is consistent with the bibliographic results.

Despite this positive effect of high whey protein intake on lean body mass, we did not detect a differential effect on stimulation of muscle protein synthesis rates of high whey protein diet vs normal protein diet. To our knowledge, there was no similar data obtained after 6 months of chronic high protein intake in the rat. However, some studies analyzed the short term effect of a test meal on muscle protein synthesis rates in the old rat. It was shown that the stimulation by feeding was altered in old rats [3], [27], [54], and that this response could be restored by increasing meal leucine [9], [27], [54], or amino acid [55] content. Similar results were described in humans, with an altered response of muscle protein synthesis to feeding in elderly subjects, and the necessity to increase leucine or amino acid or protein intake to restore a normal response (for a review see [44]). In the present study, muscle protein synthesis was not further stimulated by the high whey protein diet, implying that in these healthy rats that were probably not totally anabolic resistant, it was already maximally stimulated with the normal protein diet.

Although there was no supplemental effect of high whey protein diet on muscle protein synthesis, our results indicate that muscle protein degradation rates might be reduced in response to high whey protein diet, whatever nutritional state. This could contribute to a better sparing of lean body mass. The effect of long term high protein diet on muscle proteolysis has been little studied. Only short term studies, analyzing the respective roles of insulin and amino acids during the transition fasting – feeding were performed [7], [56]–[61]. These studies are not directly relevant with our present study, but they show that in the post-prandial state, a higher protein intake leads to a higher post-prandial inhibition of muscle proteolysis. Such an effect could also be mediated by a higher meal leucine content as shown in young men and women consuming amino acid mixtures differing in leucine content, may be through autophagy rather than through ubiquitin-proteasome system signaling [58]. Since it is not likely that a high protein intake increases post-absorptive proteolysis (probably no change), we can postulate that mean 24 h muscle proteolysis rates are lower during chronic high protein (or leucine) intake than during normal protein intake. Our data show that overall muscle proteolysis activity, as estimated by ex-vivo measurements, could be reduced after 6 months of a high whey protein diet in old rats. This could be consistent with a low mean 24 h muscle proteolysis rate. In any case, it could contribute to the sparing of lean body mass.

In conclusion, in healthy aged rats, only an increase in whey protein intake was effective in slowing lean body mass loss. This effect was not mediated by additional muscle protein synthesis stimulation in the fed state, but seemed related with a reduction in muscle proteolysis. Despite the established positive short term effects of whey proteins on muscle anabolism, after 6 months, there was no effect of protein type on lean body mass at normal protein intake. Anti-inflammatory/antioxidant supplementation had also no effect. It remains to be tested if a longer period of feeding with 12% whey protein or with polyphenols/antioxidant supplementation would have been effective. It seems also likely that a supplementation with polyphenols/antioxidant could still be effective in case of low grade inflammation.
